# Common genetic signatures of Alzheimer’s disease in Down Syndrome

**DOI:** 10.12688/f1000research.27096.2

**Published:** 2021-10-25

**Authors:** Ayati Sharma, Alisha Chunduri, Asha Gopu, Christine Shatrowsky, Wim E. Crusio, Anna Delprato

**Affiliations:** 1 BioScience Project, PO Box 352, Wakefield, MA, 01880, USA; 2 Department of Biotechnology, Chaitanya Bharathi Institute of Technology, Hyderabad, 500075, India; 3 Institut de Neurosciences Cognitives et Intégratives d'Aquitaine, CNRS UMR 5287, Pessac, 33615, France; 4 Institut de Neurosciences Cognitives et Intégratives d'Aquitaine, University of Bordeaux, Pessac, 33615, France

**Keywords:** Alzheimer’s Disease, Down Syndrome, Behavior, Memory, Learning

## Abstract

**Background:** People with Down Syndrome (DS) are born with an extra copy of Chromosome (Chr) 21 and many of these individuals develop Alzheimer’s Disease (AD) when they age. This is due at least in part to the extra copy of the APP gene located on Chr 21. By 40 years, most people with DS have amyloid plaques which disrupt brain cell function and increase their risk for AD. About half of the people with DS develop AD and the associated dementia around 50 to 60 years of age, which is about the age at which the hereditary form of AD, early onset AD, manifests. In the absence of Chr 21 trisomy, duplication of APP alone is a cause of early onset Alzheimer’s disease, making it likely that having three copies of APP is important in the development of AD and in DS.

**Methods:** We investigate the relationship between AD and DS through integrative analysis of genesets derived from a MeSH query of AD and DS associated beta amyloid peptides, Chr 21, GWAS identified AD risk factor genes, and differentially expressed genes in individuals with DS.

**Results:**  Unique and shared aspects of each geneset were evaluated based on  functional enrichment analysis, transcription factor profile and network interactions. Genes that may be important to both disorders in the context of direct association with APP processing, Tau post translational modification  and network connectivity are ACSM1, APBA2, APLP1, BACE2, BCL2L, COL18A1, DYRK1A, IK, KLK6, METTL2B, MTOR, NFE2L2, NFKB1, PRSS1, QTRT1, RCAN1, RUNX1, SAP18 SOD1, SYNJ1, S100B.

**Conclusions:** Our findings confirm that oxidative stress, apoptosis, inflammation and immune system processes likely contribute to the pathogenesis of AD and DS which is consistent with other published reports.

## Introduction

Amyloids are peptide or protein aggregates that form from the misfolding of normally soluble proteins, which then aggregate together due to their chemical properties and accumulate in extracellular compartments and organs ^4^. Amyloids form fibrous structures and plaques that are highly insoluble, resistant to degradation, and are involved in several diseases such as Alzheimer’s disease (AD), Down syndrome (DS), spongiform encephalopathies, and type II diabetes ^5, 6^. The amyloid plaques associated with AD are formed from peptides derived from the mis-processing of APP, a protein that is expressed in neurons where it is processed to Aβ peptides, some of which are found in plaques ^5, 7^. The Aβ42 peptide is preferentially deposited in plaques. The toxic peptide fragments are called beta amyloids. In AD, the amyloid plaques deposit in brain tissue, destroy neuronal connectivity, disrupt signaling at synapses, and eventually result in nerve cell death, tissue loss, and a reduction in brain mass ^5^. Oligomeric forms of the beta-amyloid and not the beta-pleated sheets in plaques themselves trigger the immune system and inflammatory processes ^8^.

Early onset AD that runs in families is linked to the *APP* and *PSEN1*/ *PSEN2* genes ^9^. A mutation in one of these three genes may cause AD to develop early whereas the more general form of the disease, late onset, is typically linked to the *APOE* gene ^10^. PSEN1/PSEN2 are transmembrane proteins that are the catalytic subunit of gamma secretase, the enzyme responsible for cleaving APP. Mutations in the *PSEN* genes may result in the abnormal cleaving and processing of APP to smaller toxic beta amyloid fragments which aggregate and accumulate ^11, 12^.

People with Down Syndrome (DS) are born with an extra copy of chromosome 21 (Chr 21) and many of these individuals develop AD as they age ^13^. This is due at least in part to the extra copy of the *APP* gene located on Chr 21. By the age of 40, most people with DS have amyloid plaques which disrupt brain cell function and increase their risk for AD ^6^. At least half of the people with DS develop AD and the associated dementia around 50 to 60 years of age, which is about the age at which the hereditary form of AD, early onset AD, manifests ^1^. Results from a longitudinal study report this number to be greater than 90%. ^14^

Duplication of *APP* alone, in the absence of Chr 21 trisomy, is another cause of early onset AD ^15, 16^ making it likely that having three copies of *APP* is important in the development of AD in DS. However, it is unclear whether the formation of excess amyloids is biologically involved in Down Syndrome itself and not just AD in DS.

In both early and late onset AD the clinical symptoms include dementia, memory decline, and the inability to retain recent information or store new memories ^17^. As the disease progresses, people with AD may exhibit problems with language, reasoning, decision making, executive function, mood swings, aggressive behavior, and apathy. Late stage symptoms of AD may result in seizures, hypertonia myoclonus, incontinence, and mutism ^18^. Death commonly occurs from general inanition, malnutrition, and pneumonia.

Memory loss and forgetfulness, which is typical in individuals with AD, is less pronounced in people with both DS and AD. This may in part be a floor-effect due to the memory deficit already present in individuals with DS ^19– 21^. Studies report an impairment in verbal short-term memory (example: serial order of a list of words) relative to visuo-spatial memory (manual selection in serial order of locations) and deficits in explicit long-term memory ^22^. Also in individuals with DS there is evidence of hippocampal dysfunction and deficits in prefrontal systems as compared with mental age-matched controls ^23^. In people with DS, the hippocampal volume is reduced prior to the onset of dementia, and these reductions were found to relate to memory mainly due to the loss of neurons and neuronal volume as a result of neurofibrillary tangle formation ^24^.

In this study we investigate the relationship between AD and DS through integrative geneset analysis of genes derived from peptides associated with amyloid plaques found in individuals with AD and DS, Chr 21 genes, AD risk factor genes, and differentially expressed genes (DEX) identified through a transcriptome analysis of people with DS for both the dorsal frontal cortex (DFC) and cerebellar cortex (CBC).

## Methods

### Geneset characteristics

All genesets used in this study are presented in *Extended data* Workbook 1 ^25^. The Chr 21 geneset was obtained from NCBI Gene. A total of 250 unique gene IDs were obtained at the time of manuscript preparation (September 1st, 2020). The AD-DS geneset, consisting of 292 genes, was obtained from GeneWeaver using “Alzheimer’s Down Syndrome” as the search term. The geneset was originally generated via gene2mesh v.1.1.1 (updated: 2019-01-07) from Medical Subject Headings (MESH Terms) GS236695 • [MeSH] Amyloid beta-Peptides: D016229. The AD risk factor geneset is comprised of 279 genes, many of which were identified and/or confirmed through a large scale GWAS of 71,880 clinically diagnosed AD and AD-by-proxy cases and 383,378 controls ^26^.

The DEX genesets for the DFC (842) and CBC (570) were obtained from The Down Syndrome Developmental Brain Transcriptome database. Human Brain Transcriptome, Department of Neurobiology Yale University School of Medicine which is a publicly accessible database containing searchable differential gene expression information of transcriptome data in developing and adult DS versus control human brains. The data was generated from 15 sets of a DS and a matched control brain each. The specimens ranged from embryonic development to adulthood ^27^.

### Geneset overlap

Common genes among the AD-DS, Chr 21, DEX DFC, DEX CBC and AD risk factor genesets were assessed using venn diagram analysis ( http://www.interactivenn.net/) and visualized with the UpSet Library in RStudio, R Version 4.0.2.

### Keyword categories

Keyword categories were used to evaluate the genesets. The keyword categories were chosen based on the major phenotypes associated with AD and DS. The terms used were: aging, Alzheimer's disease, amyloid; apoptosis, behavior cholesterol, circadian, cognition; Down Syndrome, face, fibril, immune, inflammation, insulin, learning, leptin, memory, muscle, myelin; obesity, sleep, speech, and tau.

### Functional analyses

Gene ontology characterization of the genesets was performed in both DAVID and the Gene Ontology database for Biological Process (BP). The Benjamini corrected P-value was used to determine enrichment significance. Functional information based on GO annotations for the genes associated with a keyword search term related to AD and DS were identified and noted.

Gene Ontology pathway enrichment was used to further characterize the AD-DS and Chr21 genesets in order to obtain a broader overview of collective gene function. The Benjamini-corrected P-value was used to determine significance.

### APP protein interaction network

The APP protein-protein interaction network was built in STRING (version 11.0), based on experimentally validated interactions. The combined scores for the interactions are computed by combining the probabilities from the different evidence channels and corrected for the probability of randomly observing an interaction. First and 2nd shell interactions are included in the network. The network was exported from STRING and analyzed in Cytoscape (version 3.7). Network bottlenecks and clusters were identified with Cytoscape plugins CytoHubba (version 0.1) and MCODE (version 1.6.1), respectively.

## Results

### Geneset overlap

**Figure 1.**
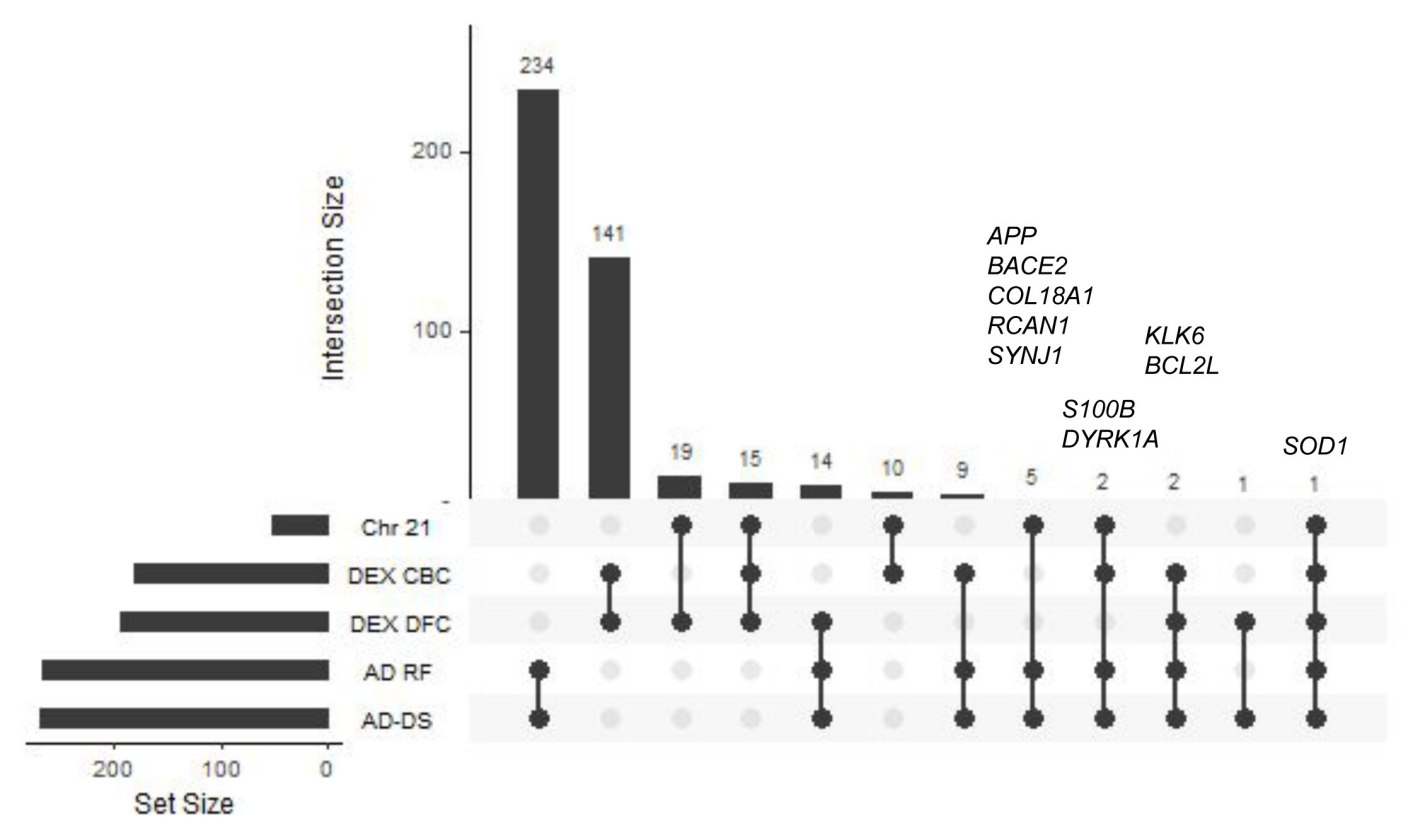
GeneSet overlap. UpSet plot showing geneset overlap highlighting gene content similarity between the AD-DS, Chr21, AD risk factors DEX DFC, and DEX CBC genes.

The number of common genes among all of the 5 genesets (AD-DS, Chr 21, AD risk factors, DEX DFC, and DEX CBC) along with the gene names and Gene Ontology classifiers are shown in Figure 1 and *Extended data* Workbook 2 ^28^. The AD-DS, Chr 21 and AD risk factor genesets overlap by eight genes: *APP*, *BACE2*, *COL18A1*, *DYRK1A*, *RCAN1*, *SOD1*, *SYNJ1*, and *S100B* ( Figure 1). *BACE2* encodes an integral membrane glycoprotein that cleaves the APP protein into amyloid-β, a critical step in the cause of AD and DS. *COL18A1* encodes the alpha chain of type XVIII collagen. It is associated with vascular deposits and senile plaques in AD brains ^29^. The *DYRK1A* gene product can phosphorylate APP and alter the protein’s stability and the formation of amyloid-β ^30, 31^. Increased *RCAN1* expression is associated with neuronal death and Tau hyperphosphorylation, as well as neurofibrillary tangle formation in individuals with DS and AD ^32^.

*SOD1* is the only gene present in all of the genesets. *SOD1* is associated with apoptosis and oxidative stress ^33^. The extra copy of *SOD* on Chr 21 results in increased gene expression and increased production of H _2_O _2_ which is believed to underlie many of the DS-related pathologies ^33^. *SOD1* is also associated with neurodegeneration in amyotrophic lateral sclerosis and AD ^34, 35^. *SYNJ1* encodes a lipid phosphatase that is involved in autophagosomal/endosomal trafficking and synaptic vesicle recycling. Its dysfunction has been linked to several neurodegenerative diseases, including AD and DS ^36^. *S100β* belongs to a family of cytokines that are strongly associated with activity underlying AD related pathologies such as APP processing, protein inclusion formation, and Tau post-translational modifications. *S100β* is also linked to DS. *S100β* levels are increased in neuronal progenitor cells of DS patients ^37^ and in human induced pluripotent stem cells derived from DS patients ^38^. Two additional genes, *KLK6* and *BCL2L*, are shared among the AD-DS, AD risk factors, DEX DFC and DEX CBC genesets. *KLK6* has been proposed as a biomarker for AD ^39^. *BCL2L* is located on the outer mitochondrial membrane and is a negative regulator of apoptosis ^40^.

### Keyword enrichment

**Figure 2.**
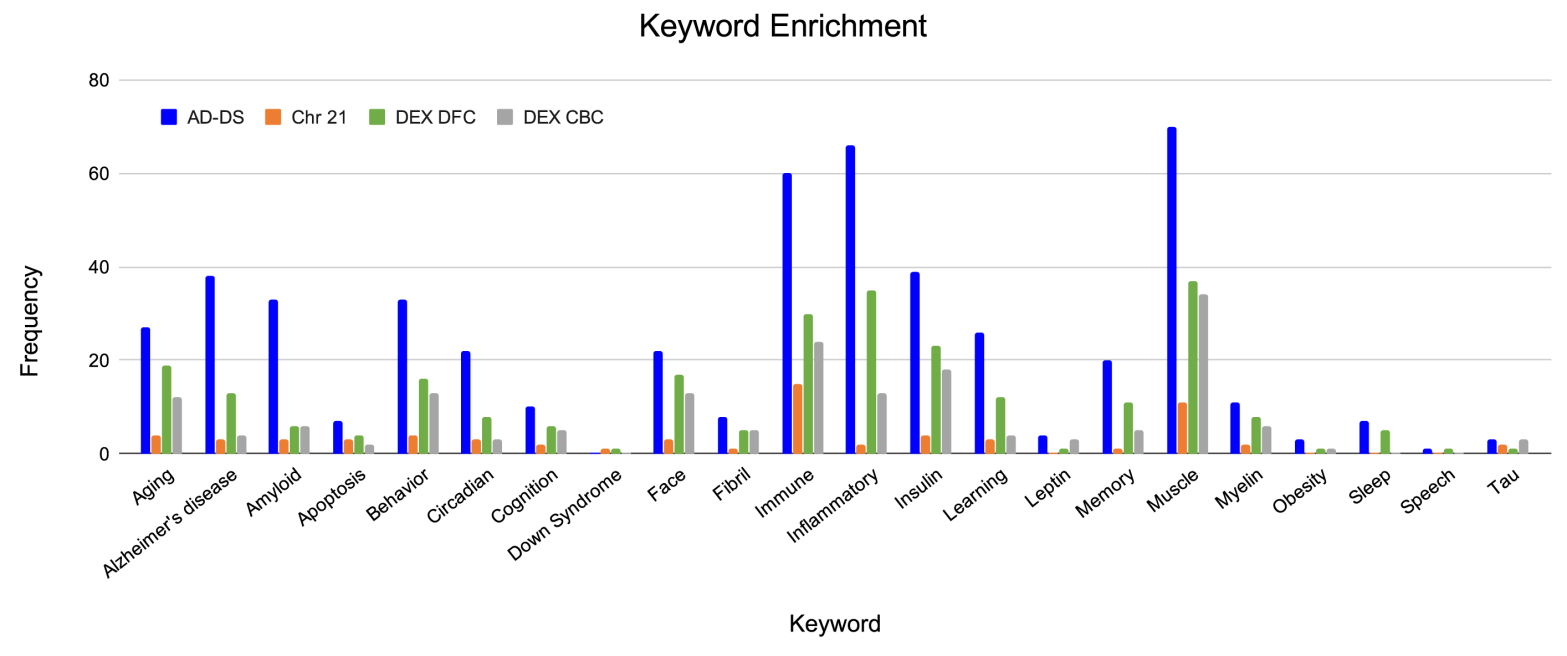
Keyword enrichment. Identification of genes associated with terms relating to AD and DS based on gene ontology term classification. AD-DS genes: blue, Chr 21 genes: orange, DEX DFC genes: green, DEX CBC genes: gray, X-axis, keyword categories; Y-axis, frequency of occurrence in the geneset.

Each of the genesets were evaluated for association with AD and DS related phenotypes ( Figure 2 and *Extended data*, Workbook 3 ^41^). The keyword categories shared among all genesets are muscle, immune, insulin, glucose, behavior, oxidation and heart. The AD-DS geneset has a high frequency of genes associated with most of the keyword categories. The largest represented categories are: AD, muscle, inflammation/immune system, insulin, amyloid, behavior, aging, learning/memory, circadian processes and face/facial features. There were no genes directly associated with DS. For the Chr 21 geneset, unlike the AD-DS geneset, there were very few genes associated with the keyword categories. The highest frequency categories are immune, muscle, aging, behavior and insulin. Three genes are connected to AD ( *NDUFV3*, *APP* and *BACE2*) and one with DS ( *DSCAM*).

The enriched keyword categories for the DEX DFC are very similar to the results obtained for the AD-DS geneset: muscle, inflammation/immune system, insulin, aging, face/facial features, behavior, AD, and learning/memory. There are 13 genes directly associated with AD ( *NDUFS2*, *APAF1*, *BACE1*, *CACNA1F*, *COX5B*, *COX6A2*, *GRIN1*, *GRIN2A*, *LPL*, *PLD3*, *PSEN1*, *RYR3*, *UQCRC1*) and one gene associated with DS ( *DSCR9*). For the DEX CBC geneset the most representative categories are again similar to the AD-DS geneset as well as the DEX DFC geneset: muscle, immune/inflammation, insulin, behavior, face/facial features, aging and amyloid. There are four genes directly linked to AD ( *ATP5H*, *APOE*, *APAF1*, *RYR3*). There are no genes directly associated with DS.

### Behavior-related genes

**Figure 3.**
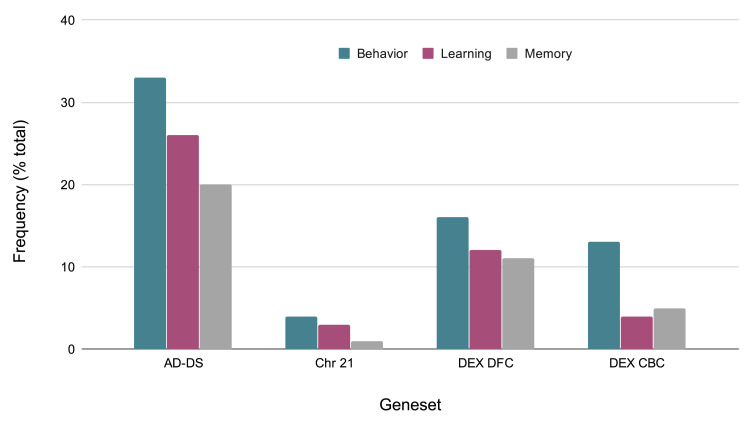
Evaluation of GO classification terms based on behavioral, memory, and learning categories. Comparison of frequencies of behavior related genes in the AD-DS, Chr 21, DEX DFC, and DEX CBC genesets. X-axis, geneset; Y-axis, frequency of occurrence (percentage of total genes) in the geneset.

Given that behavioral phenotypes are highly shared between AD and DS, the specific types of behaviors identified from the keyword enrichment were evaluated more in depth. The AD-DS geneset has a large number of behavior related genes and genes related to learning and memory: (Behavior 33, Learning 26, Memory 21). This observation is based on the GO results obtained for three random genesets of the same size: Behavior 7,2,1; Learning 0,1,1; Memory: 0,0,1. The behavior gene categories are diverse and include fear, locomotion, eating and feeding, addiction related (nicotine, cocaine, ethanol), social, and others such as circadian, mating, and response to pain. The learning categories include visual learning, associative learning, and also olfactory, motor, and nonassociative learning. The memory related categories are short-term and long-term memory, and in one instance, susceptibility to memory impairment ( Figure 3).

### Functional analyses

Many of the significant BP enrichment classifiers for the AD-DS geneset are associated with cell death (P=3.01E-83,) apoptosis (P=1.30E-70) and inflammation/immune system (P= 1.65E-36). For the Chr21 geneset, the significant BP enriched terms are linked to keratin (keratinization, P=1.04E-37), skin (skin development (P=2.83E-29) and epithelium processes (P=3.19E-15) as well as tissue (P= 3.56E-14), organ (P=3.40E-09) and anatomical structure development (P=8.66E-09). The significant pathways associated with the AD-DS geneset are related to neurodegenerative disorders (AD P=3.1E-23, Parkinson’s disease (P=1.39E-04) and Huntington’s disease P=1.36E-07) as well as many signaling pathways linked to insulin (P=1.86E-09) and inflammation (Jak/Stat P=9.49E-04), Toll receptor (P=4.04E-10), Interferon-gamma signaling (P=8.90E-06). There were no significant pathways associated with Chr 21. All pathways are listed in *Extended data* Workbook 4 ^42^.

### Transcriptional profile

The transcription profiles of the AD-DS and Chr 21 genesets were evaluated here and compared with the DEX genesets which were previously evaluated by Olmos-Serrano *et al*. ^27^ ( *Extended data*, Workbook 5 ^43^). There are 64 transcription factors and genes known to impact transcription present in the AD-DS geneset. Several of these are directly associated with AD ( *GSK3B*, *IL1B*, *MAPK3/8/10/14*, *WNT1*, *WNT3A*, *KAT5 NOTCH1* and *TNF*), Tau ( *GSK3B* and *CLU)* and amyloid ( *CD36*, *NLRP3*, *CLU*, *FOXO3*, *PARP1*, *PRNP)*. Of these, many are related to mitochondria processes ( *AKT1*, *CLU*, *GSK3B*, *HIF1A*, *MAPK*3,8,10,14, *MTOR*, *NFKB1*, *PPARGC1A*, *PARP1*, *PRNP*, *PRKCA*, *SIRT1*, *STAT3*, *SREBF2*, *UBB)* and also inflammation, oxidative stress, and aging ( *TP53*, *STAT1/3*, *NFKB1*, *HIF1A*, and *NEF2L2*).

For the Chr 21 geneset, 18 transcription factors were identified. *RUNX1* which is associated with ossification ^44^ and nervous system development ^45^ observed comparable expression in a study comparing AD and DS brains. Gene variants of *RUNX1* are associated with both AD and DS ^46^. The *OLIG1*/ *OLIG2* transcription factors regulate oligodendroglial differentiation and myelination and neuron fate commitment ^47^. In DS, due to the gene triplication, *OLIG1*/ *OLIG2* causes alterations in brain development ^48^. *OLIG2* is associated with the psychotic symptoms of AD and also schizophrenia ^49^. Of the Chr 21 transcription factors, only one is associated with mitochondria— *GABPA*— which is involved in the activation of cytochrome oxidase expression and nuclear control of mitochondrial function ^50^.

There is one common transcription factor between the AD-DS and DEX-DFC genesets: *NFE2L2* (also known as *NRF2)*, which is associated with the oxidative stress response with aging, spatial learning, memory, and neuro-inflammmation via regulation of antioxidant response elements ^51, 52^. *NFE2L2/NRF2* regulates *BACE1*, the rate-limiting enzyme for amyloid-β peptide (Aβ) generation. *NRF2* activation decreases production of *BACE1* and *BACE1* antisense RNA ( *BACE1-AS)* transcripts and Aβ production and ameliorates cognitive deficits in animal models of AD ^53^. Depletion of *NFE2L2/NRF2* increases *BACE1* and *BACE1-AS* expression and Aβ production and worsens cognitive deficits ^54^.

There are two transcription factors common between the AD-DS and DEX-CBC genesets. *MTOR* has been identified as a key target for therapeutic intervention in AD because of its regulation of several key signaling pathways: phosphoinositide 3-kinase (PI3-K)/protein kinase B (Akt), glycogen synthase kinase 3 [GSK-3], AMP-activated protein kinase (AMPK), and insulin/insulin-like growth factor 1 (IGF-1) ^55^. Both upstream and downstream components of mTOR signaling are associated with AD progression and pathogenesis. *MTOR* inhibits autophagic processes and contributes to amyloid β-peptide generation and/or clearance ^56^. *MTOR* activation also contributes to aberrant hyperphosphorylated tau ^57^. The other common TF is *NFKB1* which is a key regulator of innate immunity and strongly associated with the inflammatory response involving cytokines and chemokines ^58^. *NFKB1* is also linked to aging and AD ^59, 60^.

### APP protein-protein interaction network

An *APP* protein-protein interaction network was created to identify genes from the genesets evaluated in this study that are connected to *APP* through 1st and 2nd shell interactions. A total of 362 proteins make up the network ( *Extended data* Workbook 6 ^61^).

**Figure 4.**
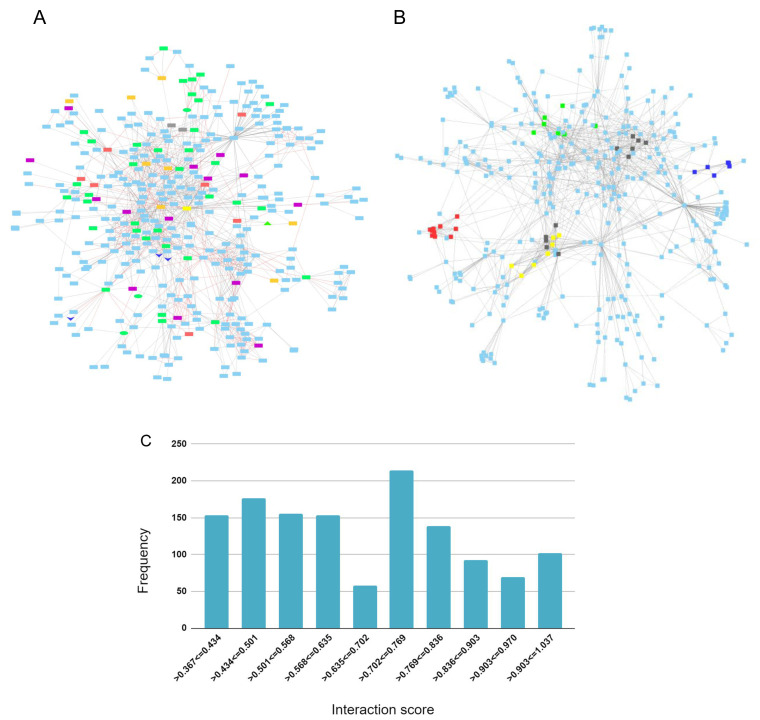
APP protein-protein interaction network. ( **A**) Geneset overlap between 1 ^st^ and 2 ^nd^ shell interactions and the AD-DS, Chr 21, DEX DFC, and DEX CBC genesets. AD-DS genes unique: red; Chr 21 genes unique: gray; DEX DFC genes unique: purple; CBC genes unique: orange; AD risk factors (RF) and AD-DS genes shared: green; DEX DFC genes shared with RF & AD-DS genes: green oval; CBC and DFC shared genes: dark blue V; CBC genes shared with RF: green triangle; APP: yellow rectangle. ( **B**) Interaction network gene clusters. Cluster 1: red – COP subunits, signalosome complex, development; Ubiquitin, Cluster 2: yellow – Tubulin, microtubules, motors, intracellular transport; Cluster 3: green – apoptosis, insulin signaling, ubiquitin, VEGFR growth factor signaling; Cluster 4: blue – Ubiquitin, autophagy; and Cluster 5: black – APP processing (PSEN, gamma secretase complex). ( **C**) Distribution frequency for interaction score.

The APP protein interaction network overlaps by 48 genes with the AD-DS geneset, 41 with the AD risk factor geneset, 21 with the DEX DFC, 12 with the DEX CBC geneset and four with the Chr 21 geneset. The shared genes are highlighted in the network to visualize and forecast additional genes that are potentially involved in APP signaling and that are relevant to both AD and DS ( Figure 4A). The top proteins that bridge (bottlenecks) the different sections of the network and that may signify information flow are: APP, ENSG00000259680 (a novel protein coding gene with similarity to immunoglobulin heavy chain variable region.), SHC1, DLG4, STUB1, KLC1, GFA1, CENPJ, and GNO1.

The APP network contains six major clusters ( Figure 4B). Cluster 1: COP subunits, signalosome complex, development, ubiquitin; Cluster 2: TUBULIN, microtubules, motors, intracellular transport; Cluster 3: apoptosis, insulin signaling, ubiquitin, VEGFR growth factor signaling; Cluster 4: UBIQUITIN, autophagy; Cluster 5: APP processing (PSEN, gamma secretase complex); and Cluster 6: TUBULIN, microtubules.

The AD risk factor genes, Chr 21, and AD-DS genes are mostly dispersed throughout the network but a couple of areas in the network contain several connected AD risk factor genes. Predicted genes of interest based on their connectivity to these areas are *METTL2B* (tRNA methylation), *IK* (immune response), *SAP18* (RNA splicing), *QTRT1* (tRNA modification), *APLP1* (Prion pathway), *PRSS1*( proteolysis, extracellular matrix digestion), *ACSM1* (lipid metabolism), *APBA2* (binds beta amyloid, synaptic transmission, and nervous system development). The validity of all of the interaction scores range from 0.4–1.00 and, for the most part, are uniformly distributed with 695 of the interactions falling in the low to mid-range of 0.4 and 0.7 and 617 falling in the mid to high-range of 0.7 and 1.0 ( Figure 4C).

## Conclusion

Genesets associated with AD, DS, and Chr 21 were evaluated to identify genes, transcription factors, and pathways that may shed light on the relationship between AD and DS. Genes common to multiple genesets are either directly involved in APP processing or in TAU post translational modification. Many of the genes associated with the amyloid plaques in AD and DS function in learning and memory. A network analysis of APP protein-protein interactions was used to analyze the topology and connectivity of the genesets and, based on interactions with common AD-DS genes and AD risk factor genes, provide the foundation to predict potential genes of interest. Genes that connect the network and represent information flow as well as regions of high interconnectivity are also of interest for follow up studies. Given the central role of APP related processes in the pathology of AD and DS, all of the proteins in the APP interaction network are either potential risk factors for AD or may contribute to disease progression in both AD and DS. Taken together, our findings confirm that oxidative stress, apoptosis, inflammation and immune system processes likely contribute to the pathogenesis of AD and DS which is consistent with other published reports ^1– 3^.

## Data availability

### Underlying data

All data underlying the results are available as part of the article and no additional source data are required.

### Extended data

Figshare: Extended Data Workbook 1. Genesets: AD-DS, Chr 21, AD risk factors, DEX DFC and CBC, https://doi.org/10.6084/m9.figshare.13106693.v1 ^25^.

Figshare: Extended Data Workbook 2. Common Genes: Gene overlap between the AD-DS, Chr 21, AD risk factors, DEX DFC and CBC genesets, https://doi.org/10.6084/m9.figshare.13106741.v1 ^28^.

Figshare: Extended Data Workbook 3. Keyword Gene Enrichment: Enrichment of the AD-DS, Chr 21, AD risk factors, DEX DFC and CBC genesets, https://doi.org/10.6084/m9.figshare.13106750.v1 ^41^.

Figshare: Extended Data Workbook 4. GO Terms and Pathways: Gene Ontology Biological Process terms and pathways associated with the AD-DS and Chr 21 genesets, https://doi.org/10.6084/m9.figshare.13106762.v1 ^42^.

Figshare: Extended Data Workbook 5. Transcription Factors: TFs present in the AD-DS, Chr 21 genesets, https://doi.org/10.6084/m9.figshare.13106774.v1 ^43^.

Figshare: Extended Data Workbook 6. APP Network File: APP protein-protein interaction network, https://doi.org/10.6084/m9.figshare.13106777.v1 ^61^.

Data are available under the terms of the Creative Commons Attribution 4.0 International license (CC-BY 4.0).
