# Supplementary material for: Common genetic signatures of Alzheimer’s disease in Down Syndrome
Source: F1000Res. 2021 Oct 25;9:1299. Originally published 2020 Nov 5. [Version 2] doi: 10.12688/f1000research.27096.2 (PMC7871416; doi:10.12688/f1000research.27096.2)
Supplement: Peer review history

# Peer review: Reviewer response for version 1

**DOI:** 10.5256/f1000research.29930.r78528

**Published:** 2021-03-09

**Authors:** Joseph H. Lee, Nicole Schupf

**Affiliations:** 1 Gertrude.H. Sergievsky Center and Taub Institute for Research on Alzheimer’s Disease and the Aging Brain, College of Physicians and Surgeons, Columbia University, New York City, NY, USA; 2 Columbia University Medical Center, New York, NY, USA

This paper by Delprato, Sharma, and colleagues examined genetic and transcriptomic factors that may explain Alzheimer’s disease in Down syndrome by putting together five genesets: genes on chromosome 21; genes identified from the search term “Alzheimer’s Down syndrome” using GeneWeaver; genes identified from a large-scale genome-wide association study (GWAS); genes identified from the differential gene expression study of the dorsal frontal cortex (DFC) and cerebellar cortex (CBC) using the Down Syndrome Developmental Brain Transcriptome database. Subsequently, they performed a series of bioinformatic analyses to generate associated genes and concluded that oxidative stress, apoptosis, and inflammation/immune system processes were important biological processes in AD in DS. This paper is timely as an increasing number of genomic and transcriptomic studies are being carried out to better understand the biology of AD in adults with DS and the non-DS population. However, there are a few points that need to be clarified:

- The title, “ Common genetic signatures of Alzheimer’s disease in Down Syndrome,” suggests that this paper deals with genes that are likely to be involved in the neurodegenerative disease process in adults with Down syndrome. However, it is stated that the differential gene expression geneset was obtained from the Down Syndrome Developmental Brain Transcriptome Database, which included 15 sets of DS brains and matched control brains, where some of the brain specimens in the database were in embryonic stages. It is unclear how well these DS brain samples from embryonic stages would represent the neurodegenerative processes associated with AD in DS. It would be informative to repeat the analysis after excluding tissues that were from embryonic stages, if the sample size is sufficient. 
- In the Introduction (page 3, lines 4-8), it is stated that “Amyloids form fibrous structures and plaques … are involved in several diseases, such as Alzheimer’s disease (AD), *Down Syndrome (DS)*, ….” Most would agree that adults with DS have a high risk of developing AD. However, it is unclear whether the formation of excess amyloids is biologically involved in the Down Syndrome itself, not just AD in DS. It should be clarified whether the wide range of DS phenotypes shown in Figure 2 can be attributed to the amyloid form fibrous structure and plaques. 

- Comparisons of the genes identified from AD-DS vs. DEX DFC and AD-DS vs. DEX CBC were interesting. It would be equally interesting to present DEX DFC vs. DEX CBC comparisons as they may represent genes involved in cognitive vs. motor processes.  

Is the work clearly and accurately presented and does it cite the current literature?

Yes

If applicable, is the statistical analysis and its interpretation appropriate?

Yes

Are all the source data underlying the results available to ensure full reproducibility?

Yes

Is the study design appropriate and is the work technically sound?

Yes

Are the conclusions drawn adequately supported by the results?

Partly

Are sufficient details of methods and analysis provided to allow replication by others?

Yes

Reviewer Expertise:

Human genetics, Genetic epidemiology, Alzheimer’s disease, Down syndrome, Autosomal dominant forms of Alzheimer’s disease.

We confirm that we have read this submission and believe that we have an appropriate level of expertise to confirm that it is of an acceptable scientific standard, however we have significant reservations, as outlined above.

# Response

**Published:** 2021-10-19

**Authors:** Anna Delprato

**Affiliations:** BioScience Project, USA

Reviewer 4:

 APPROVED WITH RESERVATIONS

 This paper by Delprato, Sharma, and colleagues examined genetic and transcriptomic factors that may explain Alzheimer’s disease in Down syndrome by putting together five genesets: genes on chromosome 21; genes identified from the search term “Alzheimer’s Down syndrome” using GeneWeaver; genes identified from a large-scale genome-wide association study (GWAS); genes identified from the differential gene expression study of the dorsal frontal cortex (DFC) and cerebellar cortex (CBC) using the Down Syndrome Developmental Brain Transcriptome database. Subsequently, they performed a series of bioinformatic analyses to generate associated genes and concluded that oxidative stress, apoptosis, and inflammation/immune system processes were important biological processes in AD in DS. This paper is timely as an increasing number of genomic and transcriptomic studies are being carried out to better understand the biology of AD in adults with DS and the non-DS population.

  However, there are a few points that need to be clarified:

- The title, “ Common genetic signatures of Alzheimer’s disease in Down Syndrome,” suggests that this paper deals with genes that are likely to be involved in the neurodegenerative disease process in adults with Down syndrome. However, it is stated that the differential gene expression geneset was obtained from the Down Syndrome Developmental Brain Transcriptome Database, which included 15 sets of DS brains and matched control brains, where some of the brain specimens in the database were in embryonic stages. It is unclear how well these DS brain samples from embryonic stages would represent the neurodegenerative processes associated with AD in DS. It would be informative to repeat the analysis after excluding tissues that were from embryonic stages, if the sample size is sufficient. 

Response: 

 The aim of the study was not to investigate and compare the neurodegeneration process in DS and AD. Our aim was to analyze gene expression patterns associated with both disorders in the context of genes located on Chr 21 because individuals with DS have a third copy of this chromosome which contains the APP gene as well as other relevant genes. 

- In the Introduction (page 3, lines 4-8), it is stated that “Amyloids form fibrous structures and plaques … are involved in several diseases, such as Alzheimer’s disease (AD), Down Syndrome (DS), ….” Most would agree that adults with DS have a high risk of developing AD. However, it is unclear whether the formation of excess amyloids is biologically involved in Down Syndrome itself, not just AD in DS. It should be clarified whether the wide range of DS phenotypes shown in Figure 2 can be attributed to the amyloid form fibrous structure and plaques. 

Response:

 We have addressed this point in the introduction by stressing that APP may account for some of the DS associated phenotypes but is most likely not solely responsible for the wide range of phenotypes associated with DS.. 

 APP may account for some of the phenotypes but given the widespread alteration throughout the DS genome, an extra copy of APP is most likely not solely responsible for the multiple and varied phenotypes associated with DS.

- Comparisons of the genes identified from AD-DS vs. DEX DFC and AD-DS vs. DEX CBC were interesting. It would be equally interesting to present DEX DFC vs. DEX CBC comparisons as they may represent genes involved in cognitive vs. motor processes.  

Response:

 The DFC and CBC comparison of differentially expressed genes was performed in the original study ( Olmos-Serranoon et al. 2016)  on which the DS developmental transcriptome database was generated. These results are reported in this Table S5 

 Summarized here. The DFC Gene ontology enrichment analysis indicated potential dysregulated biological categories including transmission of nerve impulse (Benjamini-Hochberg-adjusted (BHA) p=2.9×10-8), synaptic transmission (BHA p=1.4×10-5), cell morphogenesis (BHA p=1.4×10-5), cell-cell signaling (BHA p=6.7×10-5), and axon ensheathment (BHA p=4.8×10-2) (Table S5).

 For the CBC, enriched terms for DNA, RNA processing as well as endocytosis and vesicle transport and budding.

 Also of interest, for the DFC the number of differentially expressed (DEX) genes increased in an age-dependent manner, with significantly more DEX genes emerging over postnatal development and adulthood (periods 8-14) (Figure 1D, and Table S4) whereas for the CBC the number of DEX genes remained constant.

# Peer review: Reviewer response for version 1

**DOI:** 10.5256/f1000research.29930.r77580

**Published:** 2021-02-15

**Authors:** Ann-Charlotte Granholm

**Affiliations:** 1 Knoebel Institute for Healthy Aging and the Department of Biology, University of Denver, Denver, CO, USA; 2 Karolinska Institutet, Stockholm, Sweden

This is an excellent genetic study of comparisons between AD and DS-related AD by Delprato and collaborators. Overall, this is an interesting and timely publication. The GeneSet overlay in Figure 1 is an excellent demonstration of the findings. There are only a few comments to be made and these are listed below.

1. In the abstract, last sentence, the authors state that “Our findings indicate that oxidative stress, apoptosis, and inflammation/immune system processes likely underlie the pathogenesis of AD and DS”. However, these contributors to AD in DS have already been known for quite some time, albeit maybe not at the genetic level. It would be better to reformulate this sentence to something like this: “Our findings confirm that oxidative stress, apoptosis, and inflammation/immune system processes likely contribute to AD in DS – these processes have been investigated in animal models and post mortem human tissues previously but not at the human genetic level”. Or something like that.
2. Introduction, first sentence: “stick together” should be replaced with “aggregate”.
3. First paragraph of Introduction: “a protein that typically resides around nerve cells” should be replaced with “an integral membrane protein that is concentrated in synapses of neurons”.
  Introduction first paragraph: “Smaller aggregates of beta-amyloid and not the plaques themselves…” This could be more specific. For example “Oligomeric forms of the beta-amyloid, and not the beta-pleated sheets in plaques” or something like that.
4. Second paragraph of Introduction: “About half of the people with DS develop AD and the associated dementia around 50 to 60 years of age”. It is actually thought by most people now that 90% or more of those with DS developed AD – this should be referenced and corrected.
5. Throughout the text, “AD individuals” or “DS individuals” should be replaced with “people or individuals with AD or DS” – those who have either condition do not wish to be defined by their disease. This is very important.
6. Generally, people refer to DS-related AD as DS-AD and not AD-DS, since the DS condition came first.
7. Last sentence of Conclusions: it would not hurt to follow this statement with some literature citations that, indeed, confirm that these same processes have been proposed by others using both post mortem human tissue and animal models for DS. This would place these findings in the light of previous literature and acknowledge that these processes have been proposed by others and are here confirmed by gene expression studies.

Is the work clearly and accurately presented and does it cite the current literature?

Partly

If applicable, is the statistical analysis and its interpretation appropriate?

Yes

Are all the source data underlying the results available to ensure full reproducibility?

Yes

Is the study design appropriate and is the work technically sound?

Yes

Are the conclusions drawn adequately supported by the results?

Yes

Are sufficient details of methods and analysis provided to allow replication by others?

Yes

Reviewer Expertise:

Down syndrome and Alzheimer's disease.

I confirm that I have read this submission and believe that I have an appropriate level of expertise to confirm that it is of an acceptable scientific standard, however I have significant reservations, as outlined above.

# Response

**Published:** 2021-10-19

**Authors:** Anna Delprato

**Affiliations:** BioScience Project, USA

Reviewer 3:

 This is an excellent genetic study of comparisons between AD and DS-related AD by Delprato and collaborators. Overall, this is an interesting and timely publication. The GeneSet overlay in Figure 1 is an excellent demonstration of the findings. There are only a few comments to be made and these are listed below.

- In the abstract, last sentence, the authors state that “Our findings indicate that oxidative stress, apoptosis, and inflammation/immune system processes likely underlie the pathogenesis of AD and DS”. However, these contributors to AD in DS have already been known for quite some time, albeit maybe not at the genetic level. It would be better to reformulate this sentence to something like this: “Our findings confirm that oxidative stress, apoptosis, and inflammation/immune system processes likely contribute to AD in DS – these processes have been investigated in animal models and post mortem human tissues previously but not at the human genetic level”. Or something like that.

Response:

 Done.

  

- Introduction, first sentence: “stick together” should be replaced with “aggregate”.

Response:

 Done.

  

- First paragraph of Introduction: “a protein that typically resides around nerve cells” should be replaced with “an integral membrane protein that is concentrated in synapses of neurons”.

Response:

 Done.

 Introduction first paragraph: “Smaller aggregates of beta-amyloid and not the plaques themselves…” This could be more specific. For example “Oligomeric forms of the beta-amyloid, and not the beta-pleated sheets in plaques” or something like that.

 Response:

 Done.

  

- Second paragraph of Introduction: “About half of the people with DS develop AD and the associated dementia around 50 to 60 years of age”. It is actually thought by most people now that  90% or more of those with DS developed AD –this should be referenced and corrected.

Response:

 We have added this information to the introduction.

  

- Throughout the text, “AD individuals” or “DS individuals” should be replaced with “people or individuals with AD or DS” – those who have either condition do not wish to be defined by their disease. This is very important.

Response:

 Thank you for the correction. We have edited the text accordingly.

  

- Generally, people refer to DS-related AD as DS-AD and not AD-DS, since the DS condition came first.

Response:

 To the best of our knowledge each instance of the “AD-DS” designation refers to the datasets and figures generated in the study and not the condition of AD in people with DS.

  

- Last sentence of Conclusions: it would not hurt to follow this statement with some literature citations that, indeed, confirm that these same processes have been proposed by others using both post mortem human tissue and animal models for DS. This would place these findings in the light of previous literature and acknowledge that these processes have been proposed by others and are here confirmed by gene expression studies.

Response:

 The Conclusion section has been modified and references have been added.

# Peer review: Reviewer response for version 1

**DOI:** 10.5256/f1000research.29930.r76772

**Published:** 2021-02-08

**Authors:** William C. Mobley

**Affiliations:** 1 Department of Neurosciences, University of California San Diego School of Medicine, La Jolla, CA, USA

Sharma and colleagues have examined data for genetic contributions to the emergence of Alzheimer's disease (AD) in those with Down syndrome (DS). Their methods include the creation and/or mining of various genesets, including one for AD and DS derived from a MeSH query, as well as genesets representing Chromosome 21, AD risk factors from GWAS data, and differentially expressed genes in the DS cortex and cerebellum. Overlaps between genesets were examined to identify genes of interest, including tissues and biological processes, functional analyses, behavior and transcription as well an APP protein-protein interaction network. Overall, the findings confirm some expected associations and point to possible novel associations that may prove useful to those exploring the biology of AD in DS (AD-DS).

 Having stated the positives, additional comments may prove useful to enhance the appreciation of the work. First, it should be clarified that *APP* is expressed in neurons wherein it is processed to Aβ peptides, some of which are found in plaques. It is not true that Aβ peptides are due to misprocessing of APP but instead that one of its products the Aβ42 peptide is preferentially deposited in plaques. An increase in the relative levels of Aβ42 to shorter Aβ species is cited as playing a role in pathogenesis, as well it might, but Aβ42 is nevertheless a normal product of APP. Note, however, also, that Aβ42 and other Aβ species are present in toxic oligomeric complexes that are now viewed as more significant for pathogenesis than are the amyloid plaques.  A second comment concerns the section examining transcriptional profiles wherein a listing of differentially regulated genes is given. While the corresponding RNA levels corresponding to these genes differ in AD-DS versus controls only some of the genes listed encode transcription factors. I suspect the authors meant to convey that the products of these genes may impact transcription.

 In the end, this paper will be important to the extent that it drives the testing of specific hypotheses. The large amount of data that comes from such analyses may not readily suggest follow-on studies. Given the extensive work invested here, the authors might choose to highlight a small subset of findings and predict relationships that, if further supported, would more incisively speak to the biology of AD in DS.

Is the work clearly and accurately presented and does it cite the current literature?

Yes

If applicable, is the statistical analysis and its interpretation appropriate?

I cannot comment. A qualified statistician is required.

Are all the source data underlying the results available to ensure full reproducibility?

Yes

Is the study design appropriate and is the work technically sound?

Yes

Are the conclusions drawn adequately supported by the results?

Yes

Are sufficient details of methods and analysis provided to allow replication by others?

Yes

Reviewer Expertise:

Studies on the pathogenesis of AD in DS.

I confirm that I have read this submission and believe that I have an appropriate level of expertise to confirm that it is of an acceptable scientific standard.

# Response

**Published:** 2021-10-19

**Authors:** Anna Delprato

**Affiliations:** BioScience Project, USA

Reviewer 2:

 Sharma and colleagues have examined data for genetic contributions to the emergence of Alzheimer's disease (AD) in those with Down syndrome (DS). Their methods include the creation and/or mining of various genesets, including one for AD and DS derived from a MeSH query, as well as genesets representing Chromosome 21, AD risk factors from GWAS data, and differentially expressed genes in the DS cortex and cerebellum. Overlaps between genesets were examined to identify genes of interest, including tissues and biological processes, functional analyses, behavior and transcription as well an APP protein-protein interaction network. Overall, the findings confirm some expected associations and point to possible novel associations that may prove useful to those exploring the biology of AD in DS (AD-DS).

 Having stated the positives, additional comments may prove useful to enhance the appreciation of the work. 

 First, it should be clarified that APP is expressed in neurons wherein it is processed to Aβ peptides, some of which are found in plaques. It is not true that Aβ peptides are due to misprocessing of APP but instead that one of its products the Aβ42 peptide is preferentially deposited in plaques. An increase in the relative levels of Aβ42 to shorter Aβ species is cited as playing a role in pathogenesis, as well it might, but Aβ42 is nevertheless a normal product of APP. Note, however, also, that Aβ42 and other Aβ species are present in toxic oligomeric complexes that are now viewed as more significant for pathogenesis than are the amyloid plaques.  

 Response:

 We thank the reviewer for the clarification concerning the processing of Aβ peptides. We have included this information in the introduction of the revised manuscript.

 A second comment concerns the section examining transcriptional profiles wherein a listing of differentially regulated genes is given. While the corresponding RNA levels corresponding to these genes differ in AD-DS versus controls,  only some of the genes listed encode transcription factors. I suspect the authors meant to convey that the products of these genes may impact transcription. 

 Response:

 We have clarified this point in the revised manuscript. 

 In the end, this paper will be important to the extent that it drives the testing of specific hypotheses. The large amount of data that comes from such analyses may not readily suggest follow-on studies. Given the extensive work invested here, the authors might choose to highlight a small subset of findings and predict relationships that, if further supported, would more incisively speak to the biology of AD in DS.

 Response:

 There is a subset of findings highlighted in the Results section of the abstract. We have added more context to emphasize the significance of these findings.

# Peer review: Reviewer response for version 1

**DOI:** 10.5256/f1000research.29930.r74335

**Published:** 2020-11-24

**Authors:** Daniel W. Nixon

**Affiliations:** 1 Cancer Treatment Centers of America, Southeastern Regional Medical Center, Newnan, GA, USA; 2 Morehouse School of Medicine, Atlanta, GA, USA

This is a well-written paper that adds useful information to the literature. The connection between Down syndrome and Alzheimer’s disease is clinically well established and is currently not preventable, leading to increased difficulty in Down syndrome management. Genetic examination as in this paper should stimulate further molecular research in Down syndrome including the genetic influences on obesity, common in Down syndrome and a risk factor in Alzheimer's disease. I suggest further research by this group in adipokines such as leptin and adiponectin as well as interactions between Wnt, BACE, Notch, BCL and DYRK. This could lead to better understanding of the relationships between Down syndrome, Alzheimer’s disease, leukemia and solid tumors. Down syndrome is associated with an increased risk of leukemia and a decreased risk for solid tumors while Alzheimer’s disease is associated with a decreased risk for various solid tumors.

Is the work clearly and accurately presented and does it cite the current literature?

Yes

If applicable, is the statistical analysis and its interpretation appropriate?

Yes

Are all the source data underlying the results available to ensure full reproducibility?

Yes

Is the study design appropriate and is the work technically sound?

Yes

Are the conclusions drawn adequately supported by the results?

Yes

Are sufficient details of methods and analysis provided to allow replication by others?

Yes

Reviewer Expertise:

NA

I confirm that I have read this submission and believe that I have an appropriate level of expertise to confirm that it is of an acceptable scientific standard.

# Response

**Published:** 2021-10-19

**Authors:** Anna Delprato

**Affiliations:** BioScience Project, USA

Reviewer 1:

 This is a well-written paper that adds useful information to the literature. The connection between Down syndrome and Alzheimer’s disease is clinically well established and is currently not preventable, leading to increased difficulty in Down syndrome management. Genetic examination as in this paper should stimulate further molecular research in Down syndrome including the genetic influences on obesity, common in Down syndrome and a risk factor in Alzheimer's disease. I suggest further research by this group in adipokines such as leptin and adiponectin as well as interactions between Wnt, BACE, Notch, BCL and DYRK. This could lead to better understanding of the relationships between Down syndrome, Alzheimer’s disease, leukemia and solid tumors. Down syndrome is associated with an increased risk of leukemia and a decreased risk for solid tumors while Alzheimer’s disease is associated with a decreased risk for various solid tumors.

 Response:

 Thank you for the encouraging review and the information pertaining to cancer and solid tumor formation in the context of AD and DS. We will keep this in mind for future studies.
